# Population Dynamics of *Plasmodium vivax* in Mexico Determined by CSP, Pvs25, and SSU 18S rRNA S-Type Polymorphism Analyses

**DOI:** 10.3390/microorganisms13092221

**Published:** 2025-09-22

**Authors:** Lilia González-Cerón, Delfino de Jesús Gómez-Pérez, Frida Santillán-Valenzuela, Marbella Ovilla-Muñoz, Carmen Guzmán-Bracho, Angélica Pech-May, Gerardo R. Amores, Alberto Montoya-Pérez, Cuauhtémoc Villarreal-Treviño

**Affiliations:** 1 Centro Regional de Investigación en Salud Pública, Instituto Nacional de Salud Pública, 4ta Avenida Norte and 19 Calle Poniente, Tapachula 30700, Chiapas, Mexico; delfinogomezperez@gmail.com (D.d.J.G.-P.); fsantill@insp.mx (F.S.-V.); angelica.pech@insp.mx (A.P.-M.); cvilla@insp.mx (C.V.-T.); 2 Centro de Información para Decisiones en Salud Pública, Instituto Nacional de Salud Pública, Ciudad de Mexico 14080, Mexico; movilla@insp.mx; 3 Instituto Nacional de Diagnóstico y Referencia Epidemiológica, Secretaria de Salud, Ciudad de Mexico 01480, Mexico; cguzmanbracho@hotmail.com; 4 Departamento de Ingeniería Genética, Centro de Investigación y Estudios Avanzados, Irapuato 36824, Guanajuato, Mexico; amoresgr@gmail.com; 5 Centro Nacional de Diagnóstico y Referencia, Ministerio de Salud, Managua 11165, Nicaragua

**Keywords:** *Plasmodium vivax*, circumsporozoite, *pvcsp*, *pvs25*, S-type 18S SSU rRNA, polymorphism, malaria foci, Mexico, Nicaragua

## Abstract

In Mexico, *Plasmodium vivax* transmission has been confined to the northwestern and southern regions since 2000. Parasites from five malaria foci were analyzed using three genetic markers. The circumsporozoite gene was examined by PCR-RFLP and sequencing, and *pvs25* mutations and variants of ribosomal 18S SSU rRNA S-type were also determined. Previous data from the southernmost Pacific in Chiapas were included in the analysis. Both the VK210 and VK247 types of *pvcsp* were detected, and VK210 had greater haplotype diversity (0.860) than VK247 parasites (0.198). Two *pvs25* mutations (Q87K and I130T) yielded three haplotypes, and two ribosomal variants were detected. Gene and multilocus haplotype frequencies varied among malarious foci (*p* < 0.001). An AMOVA test, *F_ST_* values, and Spearman’s correlation suggested a structured *P. vivax* population among the malaria foci. Each malaria focus across the northwestern and southern regions retained a portion of the past countrywide *P. vivax* population, which seems unique in Latin America. In the Lacandon region (LR), a linkage equilibrium between *pvs25* haplotypes and the ribosomal variants within the VK247 or VK210 populations was observed. This region harbored the broadest reservoir of *P. vivax* haplotypes, and the high adaptation of parasites in the northwestern region represents a challenge for malaria elimination. These finding are relevant for monitoring and epidemiological surveillance.

## 1. Introduction

Malaria is a sickness caused by *Plasmodium* parasites and transmitted to humans by infected *Anopheles* mosquitoes. *Plasmodium vivax* is the second most prevalent species, causing high morbidity in tropical and subtropical regions worldwide. In Latin America, *P. vivax* was responsible for about 73% of the 505,600 cases reported in 2023 [1]. In Mexico, malaria case reporting began in the 1950s, with *P. vivax* cases predominating across the Mexican territory [2]. In this country, malaria cases fluctuated annually, ranging from ~3000 to ~55,000 during the 1960s–1970s period. In the 1980s, the number of cases increased steadily, reaching over 130,000 in a single year. Incidence rates per 100,000 inhabitants varied significantly among states, from 2497 in Quintana Roo in 1984 to just 0.1 in Guanajuato in 1982 [2]. Later, in the 1990s, the number of cases constantly decreased, and in 2009, 2636 *P. vivax* cases were reported [2]. Afterwards, the number of *P. vivax* cases has remained low, confined to regions with persistent malaria transmission. In 2017 and 2024, the country reported 551 and 342 confirmed *P. vivax* cases, respectively [2]. Last year, cases were reported in Chiapas, with a few others in Oaxaca and Chihuahua. Based on the WHO criteria, Mexico progressed to the pre-elimination phase in 2009 [3].

In Mexico, primaquine was introduced in the 1960s to treat *P. vivax* patients, and the large number of cases led a malaria program to implement a monthly and intermittent combined therapy of Chloroquine and primaquine [4]. This treatment, however, may have not fully eliminated all infections caused by hypnozoites, thereby potentially contributing to ongoing malaria transmission [5]. Since then, malaria incidence has decreased to few hundreds of cases, with treatment protocols typically involving combined CQ/PQ treatment with either 7- or 4-day regimens [6].

Molecular genetic studies are crucial for understanding the epidemiology, diversity, geographical distribution, and dynamics of *P. vivax* populations [7]. In Mexico’s southernmost Pacific (SMP) region, specifically in the state of Chiapas, genetic studies have been conducted using markers that encode proteins in both the sporozoite and blood stages. These studies, along with those on genes associated with drug resistance, have suggested a low-to-moderate parasite diversity with exclusive haplotypes not yet reported elsewhere on the continent [8,9,10,11,12,13]. Also, longitudinal studies revealed that the most adapted or highly frequent haplotypes of *pvama1_I–II_* and *pvmsp1*_42_ persisted across 17 years [14,15]. In this region, two circumsporozoite repeat variants were detected: VK210 and VK247. While the VK247 type was highly conserved, two VK210 types differed at the variable carboxyl terminus. One variant was related to the Sal-I strain, while the other expressed the domain ANKKAEDA at the carboxyl terminus [12].

In the SMP, high compatibility was found in sympatric *P. vivax*–vector combinations. *P. vivax* from patients living in the foothills produced high infection rates in *Anopheles* (*subgenus Anopheles*) *pseudopunctipennis*, a species abundant in that region. These parasites were low-infectious to *Anopheles* (*subgenus Nyssorhynchus*) *albimanus*, a vector of the coastal regions. In contrast, *P. vivax* from patients coming from the coastal regions were highly infectious to *Ny. albimanus* and did not produce infection in *An. pseudopunctipennis* [16]. *Pv*CSP VK210 and VK247 were partially associated with vector susceptibility [17,18]. Other studies showed that mutations in genes expressing ookinete proteins, which are involved in parasite development in the mosquito (such as Pvs25, Pvs28, SOAP, and CTRP), and the ribosomal *18S SSU rRNA S-type* variants defined two genetic lineages [16]. Strong linkage disequilibrium was observed within these lineages, which is likely a result of geographic and vector restrictions [16].

In this study, *pvcsp*, *pvs25*, and *18S SSU rRNA* were analyzed, for the first time, in different malaria foci across the northwestern and southern regions. Additionally, this study sought to determine whether *P. vivax* genotypes from the SMP were also predominant in other regions and whether a pattern could be detected regarding vector specificity. For comparisons, genetic data previously obtained from the SMP were incorporated into the analysis, and *P. vivax* samples from Central America were included as an outgroup.

## 2. Materials and Methods

### 2.1. P. vivax Samples and Geographic Origin

Three hundred and thirty-five *P. vivax* blood smear samples were obtained from the National Malaria Control Program in Mexico. The samples were previously fixed with methanol, stained with Giemsa, and diagnosed with *P. vivax* infection [19]. Table 1 indicates the number of samples per state from a period of three years: 2010–2012. This period corresponds to the early pre-elimination phase, and after malaria transmission, it was focalized. Therefore, these *P. vivax* samples might represent the most likely persistent haplotypes in this country.

Another 12 *P. vivax*-infected blood samples impregnated in Whatman #2 filter paper from the SMP, Mexico [16], and 58 samples from Central America (the Autonomous Region of the North Caribbean Coast (RACCN) in Nicaragua) [20,21] were included in the analysis since the Mesoamerican region shared similar anopheline species [16,22].

**Figure 1:**
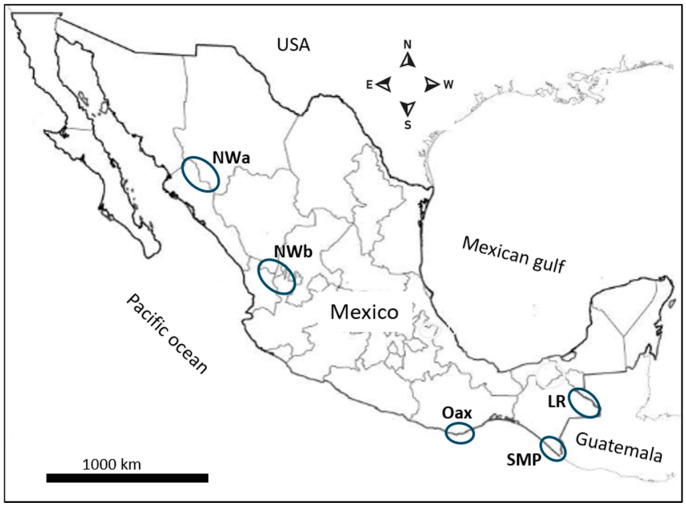
The geographical distribution of the malaria foci and source of *P. vivax* samples in Mexico. The blue circles represent the malaria foci studied here.

The spatial representation in Figure 1 delineates malaria foci as follows: NWa corresponds to the interstate regions of Sinaloa and Chihuahua, while NWb corresponds to the interstate area comprising Durango, Nayarit, and Jalisco. These are areas with difficult access where malaria risk is low, and transmission is intermittent. Malaria cases in these regions have gradually declined with some fluctuations. In Pochutla, Oaxaca (OAX) malaria transmission occurred on the coastal and foothill regions. After years of intensive control measures—which included disturbing mosquito breeding sites with community participation and patient detection and treatment—this state did not report any cases in 2014–2021. However, an outbreak was reported in 2022. These regions have ecological conditions for breeding *An. pseudopunctipennis* [23,24]. The SMP has a tropical wet climate, with a long rainy season, although no autochthonous cases are currently reported. Since 2015, most reported cases in Chiapas correspond to the Lacandon regions (LRs). The climate there is tropical wet with abundant rainfall from May to November, with annual precipitation ranging between 2500 and 3500 mm [25]. In Chiapas, the SMP and LRs are about 500 km apart, and they have historically been migration routes for people traveling to the United States from diverse origins [26]. These regions are about 1000 km away from Nicaragua, Central America.

### 2.2. Sample Preparation and DNA Extraction

Dry blood smear samples were sprayed with molecular-grade water and scraped out from a microscope slide, using the clean edge of a new glass slide. Low-density powder was meticulously transferred into a 1.5 mL Eppendorf tube. The thick smear, conversely, remained preserved on the original slide for storage. DNA extraction from the powder was conducted using the QIAmp DNA Blood Minikit (Qiagen, Redwood City, CA, USA), following the manufacturer’s protocol. Genomic DNA was eluted with 50 µL of molecular-grade water and cryopreserved at −20 °C until further analysis. From infected blood samples on filter paper (from SMP and Nicaragua), three 5 mm diameter circles from each filter paper were used to extract genomic DNA as described above.

### 2.3. Molecular Markers

Three markers positioned on different chromosomes were analyzed: *csp* variable regions (CRR and carboxyl variable terminus), a *pvs25* polymorphism at a DNA segment containing Q87K and I130T variable codons [16], and ribosomal variants.

### 2.4. PCR Amplification and Genotyping

Circumsporozoite gene (*pvcsp*). All *P. vivax* samples were screened for *pvcsp* gene amplification, which included the full central repeat region (CRR) and the variable carboxyl terminus, using primers and PCR conditions as previously reported [12]. Supplementary Figure S1 shows that the quantity of stained blood smears and parasitemia were not the sole determinants for PCR amplification success in a batch of samples. For RFLP analysis, amplified products were digested with AluI (New England Biolabs, Beverly, MA, USA) and BstOI (Promega, Madison, WI, USA) restriction enzymes as previously described [12]. Negative controls (uninfected blood samples) and positive controls (*P. vivax*-infected blood samples) were included in each run as amplification references. Furthermore, smear blood samples from different states, years, and PCR-RFLP patterns were selected for sequencing. Only isolates exhibiting *pvcsp* amplification were examined for *pvs25* and the ribosomal variants.

*Pv25* gene. A gene fragment of ~250 bp encompassing variable codons Q87K and I130T was amplified employing primers Pvs25-F23 (5′-GTG TAT GTG TAA CGA AGG GCT-3′) and Pvs25-R214 (5′-TAC CAA AAC GGG AGA AAC TG-3′). The PCR master mix composition and reaction conditions were previously reported [26].

Ribosomal *18S SSU rRNA S-type*. A segment of the *P. vivax* ribosomal was amplified using forward (SSU-F) and reverse (SSU-R) primers as previously reported [16]. The two variants were distinguished by differences in the size of the amplified product: 450 bp for rVar2 (Sal-I) and 480 bp for rVar1 (Thai) [27].

### 2.5. Sanger Sequencing

For *pvcsp* gene sequencing, groups of samples were selected at random based on their PCR-RFLP patterns and geographic origin. The *pvcsp* and *pvs25* gene segments were resolved on 1% agarose gels. Fragments of the appropriate molecular size were then purified using the Wizard Plus Minipreps DNA Purification System (Promega, Madison, WI, USA). The purified fragments were sequenced by Macrogen Inc. (Seoul, Republic of Korea), using the same forward and reverse primers that were used for amplification. The quality of pherograms was manually revised using BioEdit v7 [28], and consensus sequences were generated. The sequences were deposited in GenBank (NCBI) with accession numbers PX225108–PX225182 for *pvcsp* and PX236386–PX236514 for *pvs25*.

### 2.6. Data Analysis

Previous results obtained in the SMP for *pvs25*, *pvcsp*, and ribosomal variants were included in the analysis [13,16]. *Pvs25*: EU024410.1, EU024411.1, EU024414.1, EU024416.1, EU024417.1, EU024419.1, EU024420.1, EU024437.1, EU024438.1, EU024449, EU024455.1, EU024458.1, EU024466.1-EU024472.1 [13], MN015361-MN015369 [16]. Another 11 sequences were extracted from plasmoDB (SRP046167, SRP046170, SRP046174, SRP046175, SRP046181-SRP046184, SRP046187, SRP046194, SRP046198) [29]. The Sal-I sequence (XM_001608410.1) was employed as a reference. Nucleotide sequences were aligned utilizing CLUSTALW within BioEdit v7 [28].

For each *pvcsp* PCR-RFLP pattern, a code was assigned according to previous studies in the SMP and Nicaragua [12]. The Sal-I sequence (XM001614511.1) and others from the SMP were used to make comparisons: Mxch1-VK210a (JQ511263.1), Mxch5-VK210d (KF437876.1), Mxch4-VK210b (JQ511267), Mxch6-VK247_I (JQ511270), and Mxch13-VK210aII (JQ511280.1) [12].

For *pvs25*, genetic parameters were estimated for each malaria focus and the total sequences from Mexico, such as the number of mutations (n) and segregating sites (S), the mean number of pairwise differences (k), haplotype number (h), haplotype diversity (Hd), and both nucleotide (π) and genetic (θ) diversities. Neutrality tests, such as Fu’s Fs and Tajima’s D, were calculated to determine departure from the neutral theory of evolution, in DnaSP v5.1 [30]. To analyze the genetic differentiation among parasite groups, pairwise comparisons were made by calculating a matrix of *F_ST_*; values range from 0 (no difference) to 1 (completely isolated). The *Nm* values were also estimated as an indirect measure of gene flow based on the calculated *F*_ST_ values. To infer population history, the mismatch distribution’s sum of squared deviations (SSD) and Raggedness index were estimated by running 10,000 simulations in Arlequin v3.5.2 [31]. Low values support the expansion model, whereas high and significant values suggest a deviation from it. *Pvs25* mutations and haplotype frequencies were compared across malaria foci utilizing the χ^2^ test (Kruskal–Wallis non-parametric test) at the 95% confidence level in Stata v14.

The genetic variance for different hypothesized population groupings was estimated using an analysis of molecular variance (AMOVA) [32] for *pvs25* and multilocus haplotypes, using the distance method of Kimura 2P, with 10,000 permutations. These estimations were performed in Arlequin v3.5.2 [31]. To assess association patterns in the allele distribution of *P. vivax* from different malaria foci across Mexico, pairwise Spearman correlation analyses were conducted [33]. The analysis utilized 10 allelic variants from three molecular markers: *pvcsp*, *pvs25*, and the ribosomal variants. Procedures on binary data and visualizations were carried out using RStudio version 2023.03.1, incorporating the stats v4.4.1, corrplot v0.95, FactoMineR v2.11, and ggplot2 v3.5.2 packages. Correlations were visualized via heatmaps, with color gradients and circle size indicating direction and strength, respectively. A value of −1 denoted strong negative correlation, +1 a strong positive correlation, and 0 no association. The size of the circle encoded the absolute value of the correlation coefficient, so larger circles indicated stronger correlations regardless of sign, while smaller circles indicated weaker correlations closer to zero.

The frequencies of multilocus haplotypes were estimated per malaria focus using three markers and between the *pvs25* polymorphism and the ribosomal variants for each *pvcsp* type. To estimate linkage disequilibrium (LD) between two or three loci, LIAN was used to simulate random allele assortment at each locus. The null hypothesis (H0) VD = Ve was tested by calculating the statistical difference between the observed variance (VD) and the expected variance (Ve)—that is, LD—by computing a *p*-value using Monte Carlo (PMC) simulations with 10,000 re-samplings without replacement in LInkage ANalysis v3.7. For the I^A^_S_ index, a measure of multilocus LD, high values suggest limited recombination and clonal expansion, while low values suggest more frequent recombination [34].

## 3. Results

### 3.1. Circumsporozoite Gene Polymorphism

#### 3.1.1. PCR-RFLP of *Pvcsp*

Information regarding parasitemia, amount in smear samples, and successful *pvcsp* amplification is shown in Figure S1. For NWa, Sinaloa contributed 64.3% of the samples (three, seven, and eight from 2010, 2011, and 2012, respectively), and Chihuahua contributed 35.7% (four and six from 2010 and 2012, respectively). Meanwhile, for NWb, Durango contributed 64% of the samples (4, 3, and 34 from 2010, 2011, and 2012, respectively), Nayarit contributed 28.1% (6, 5, and 9 from 2010, 2011, and 2012, respectively), and Jalisco contributed 7.8% (5 from 2012). In 183 samples (54.3% of the total), PCR-RFLP analysis was successfully achieved (Table S1). This analysis revealed the presence of both *pvcsp* CRR types, VK210 and VK247, with their frequencies varying significantly among malaria foci in Mexico (χ^2^ = 35.8, *p* < 0.001). The VK247 type was identified in 79.8% of isolates (*n* = 222) and showed clear predominance in the NWa (92.9%, *n* = 28), NWb (100%; *n* = 64), and Oaxaca foci (100%; *n* = 28). In contrast, the LR and SMP foci showed VK247 prevalence rates of 54% (*n* = 63) and 53% (*n* = 39), respectively, with the remaining isolates being VK210. Nationwide, three VK247 types were defined by PCR-RFLP (Figure S2): VK247_I was predominant and present in all malaria foci. Another variant, VK247_III, was detected in a single isolate in NWb (sample form Nayarit state, collected in 2011), while VK247_II was exclusive to the LR (six isolates from 2012).

*Pvcsp* PCR-RFLP pattern VK210a was identified in 21 isolates, representing 11.5% of the total. Additionally, five isolates harbored VK210b and two isolates VK210d. Two distinct PCR-RFLP patterns were also detected, each in a single isolate: VK210g, which exhibited a smaller molecular size than VK210a but a similar restriction pattern, suggesting variation in the number of repeats; and VK210h, which displayed a molecular size comparable to VK210a but a different restriction pattern, suggesting polymorphism within the carboxyl variable terminus (Figure S2). Moreover, two samples demonstrated mixed genotype infections (VK210a/VK247 and VK210a/b). The highest number of genotypes were detected in the LR. The geographical distribution of *pvcsp* genotypes is delineated in Table 2.

All samples from RACCN, Nicaragua, were VK210; 95% of them had a PCR-RFLP pattern similar to VK210a, and the other three samples had a different pattern (VK210e).

#### 3.1.2. *Pvcsp* Sequence Polymorphism

Gene sequences were obtained from 75 isolates, revealing greater genetic polymorphism among VK210 sequences than was detected by PCR-RFLP. Across all sequences, the RI (KLKQP) amino acid domain, which flanks the amino-side repeat region, was conserved. Alignment was performed using the post-repeat carboxyl variable side, extending up to the GQGQ domain. Polymorphism was detected within either the repeat region, the variable carboxyl region, or both. Forty-seven VK247 sequences from different malaria foci yielded three haplotypes (as determined by PCR-RFLP and sequencing). Forty-two of the VK247_I sequences, from different malaria foci, were resolved by PCR-RFLP and had identical nucleotide sequence. Because of this, and due to limited financial resources, no further sequencing was performed. An additional four isolates showing the same PCR-RFLP VK247_III pattern were similar to VK247_I, except the deletion of one repeat at the CRR (Figure S3A). Likewise, the VK247_II sequence was distinguished from VK247_I only based on the absence of the amino acid segment GAGGQAAGGNAANKKAGDA at the carboxyl terminus (Figure S3B).

VK210 parasites displayed five distinct PCR-RFLP patterns. Nucleotide sequencing for two samples with PCR-RFLP VK210g (in LR) and VK210a (in NWa) was unsuccessful. Because gene variation was observed in the first group of VK210a sequences, the remaining VK210 samples were also sequenced. In 20 sequences classified as VK210a (resembling Sal-I), six subvariants were identified: IA (*n* = 6), IB (*n* = 4), IC (*n* = 1), ID (*n* = 1). The VK210a_II variant was identical to Mxch13 (JQ511280.1) from SMP (Figure S3A). Variant IE (*n* = 1) shares identity with IA and II and had 19 repeat units (Figure S3A).

One sample from Chihuahua (NWa) identified as VK210h had two copies of the GGNAANKKAEDA domain flanked by one copy of GGNA at the carboxyl terminus. This isolate corresponded to the ANKKAEDA domain group, along with VK210b and VK210d (Figure S3A,B). These differed in their pattern of peptide repeat motifs (PRMs) from VK210a isolates. Most of the VK210 sequences were from LR, and all VK210 sequences yielded nine haplotypes and exhibited a higher Hd (0.860) compared to the VK247 parasites (Hd = 0.198; *n* = 47).

**Figure 2:**
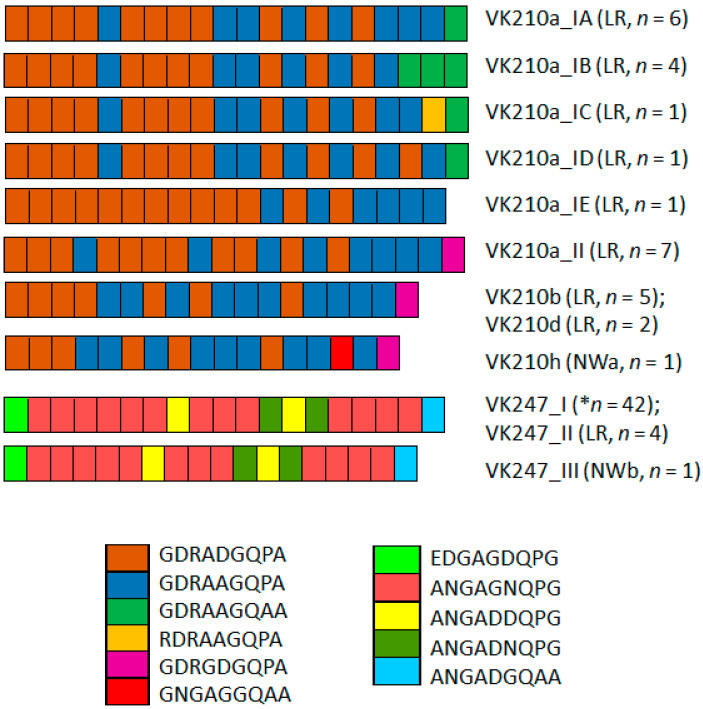
*P. vivax* circumsporozoite peptide repeat motif (PRM) variation in CRR of *P. vivax* VK210 and VK247 sequences. Two main PRM types predominated in VK210a: subtypes IA, IB, IC, and ID. Notably, last PRM in VK210a_II, VK210b, VK210d, and VK210h was different (pink). Conversely, VK210a_IE lacked this PRM. Overall, VK210 variants exhibited 2–4 distinct PRMs and VK247 5 PRMs. LR, Lacandon region; NWa, far northwestern; NWb, northwestern. * indicates that this type was from NWa, NWb, OAX, and LR.

Two PRMs predominated in the CRR VK210: GDRADGQPA and GDRAAGQPA. Other less frequent PRMs were detected in the PRM flanking the carboxyl region (Figure 2), with the exception of VK210a_IE, which had 19 repeats. In the VK247 CRR, the amino acid repeat ANGAGNQPG predominated (Figure 2).

### 3.2. Pvs25 Polymorphism and Genetic Structure

For the 129 blood smear samples from different malaria foci in Mexico (NWa, NWb, OAX, and LR), a *pvs25* sequence of 249 bp (nt 175–423) was obtained. Compared to the Sal-I strain, two polymorphisms were detected at codons 87 (cag/Q → aag/K) and 130 (atc/I → acc/T).

**Figure 3:**
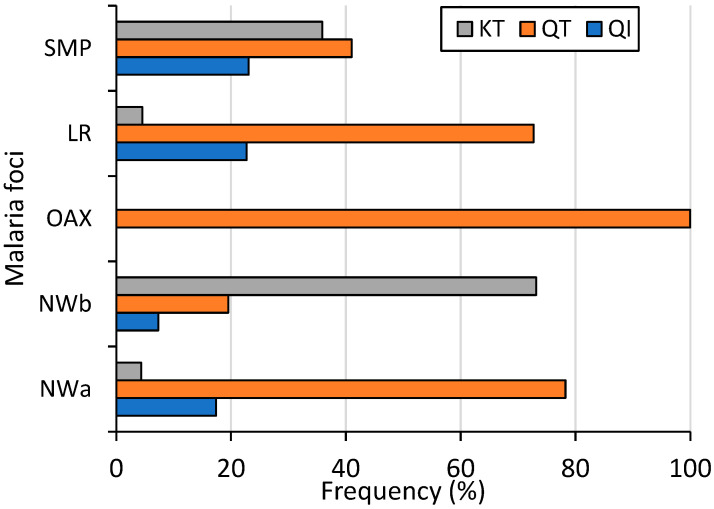
Frequency of Pvs25 haplotypes across malaria foci in Mexico (χ^2^ = 37.7, *p* < 0.001). NWa (*n* = 23), NWb (*n* = 41), OAX (*n* = 21), LR (*n* = 44), SMP (*n* = 39).

At the country level (including 39 sequences from SMP), the amino acid change Q87K was present in 28.1% of the samples. Its frequencies varied significantly among malaria foci: 4.4%, 77%, 0%, 4.3%, and 35.9% in NWa, NWb, OAX, LR, and SMP, respectively (χ^2^ = 68.5, *p* < 0.001). Conversely, the amino acid change I130T was at a high frequency (86.2%) countrywide, with no significant difference observed among foci (74–100%, χ^2^ = 4.13, *p* = 0.388). Among *P. vivax* isolates, 15.4% had haplotype QI (wild haplotype), 56.5% had QT, and 28% had KT. These three haplotypes were found in all malaria foci except in OAX, and their frequencies varied significantly (χ^2^ = 37.7; *p* < 0.001, 95% confidence) (Figure 3). The QI haplotype ranged from 0% in OAX to 23.1% in SMP; QT was found at a high frequency in four malaria foci, varying from 19.5% in NWb to 100% in OAX; and KT varied from 0% in OAX to 73.2% in NWb. One sample exhibited a mixed Q-I/T genotype infection. All 55 samples from Nicaragua had wild-type sequences (Figure 3).

Table 3 compares the genetic diversity parameters using *pvs25* among malaria foci in Mexico. SMP exhibited the highest k value, nucleotide diversity, and haplotype diversity. Conversely, the lowest diversity was detected in LR. The highest and positive Fu’s Fs and Tajima’s D values were observed in SMP, although these values were not statistically significant. In this region, the values for mismatch distribution SSD and Raggedness indexes were significant: 0.0175 (*p =* 0.04) and 0.1622 (*p =* 0.02), respectively.

The AMOVA using *pvs25* (*n* = 168) revealed that the percentage of variation was higher within populations (70%) than among malaria foci (30%). The *F_ST_* index, based on *pvs25*, was the highest between foci NWb and OAX, followed by LR or NWa. The lowest *F_ST_* index was observed between NWa and OAX (Table 4). In accordance, the lowest *Nm* values were observed between NWb and OAX or LR or NWa.

### 3.3. Frequencies of 18S rRNA S-Type Variants

**Figure 4:**
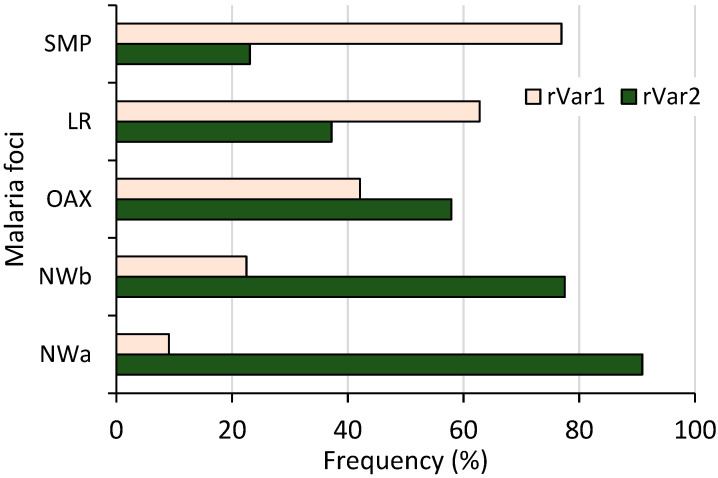
Frequency of *P. vivax* ribosomal rRNA 18S S-type variants in different malaria foci from Mexico. Both variants showed inverse frequency trend, differing from northwestern to southernmost regions (n = 163, χ^2^ = 44.12, *p* < 0.001).

Both ribosomal variants were detected; 87 and 76 had rVar2 and rVar1, respectively, and their frequencies varied among malaria foci (χ^2^ = 30.32, *p* < 0.001, 95% confidence) (Figure 4). A distinct latitudinal pattern was evident: as the frequency of one variant increased, the other’s decreased from the northwestern to southern foci. Specifically, the rVar2 variant progressively declined from 90.1% in NWa to 23.1% in SMP. Variant rVar1 had its highest frequency in SMP (76.9%) before gradually decreasing to 9.1% in NWa. All 55 samples from Nicaragua had the rVar2 variant.

### 3.4. P. vivax Multilocus Haplotype Analyses

**Figure 5:**
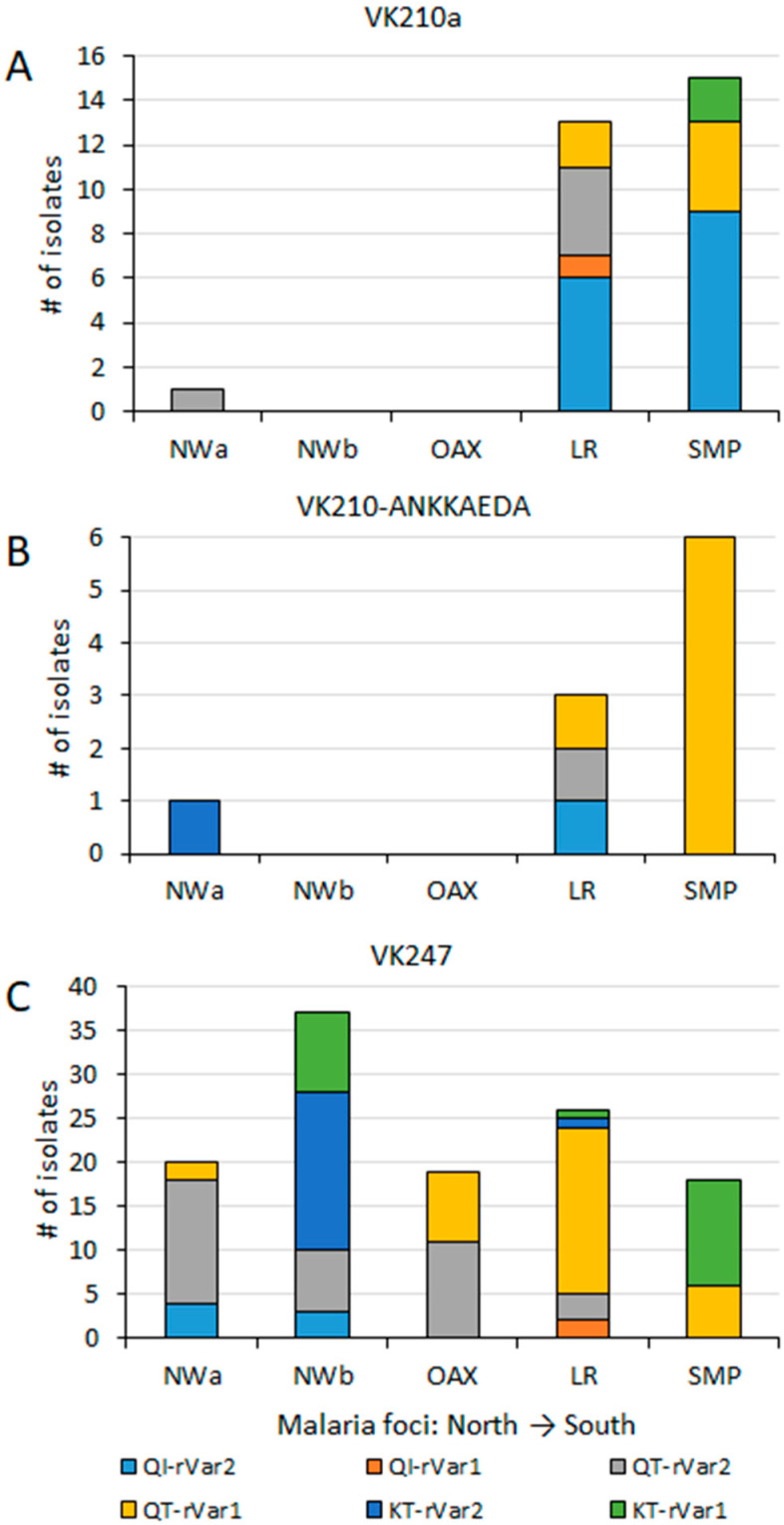
Geographical distribution of haplotypes by *pvcsp* type, *pvs25*, and ribosomal variants across malaria foci in Mexico. *Pvs25*, and ribosomal variants haplotypes are indicated per *pvcsp* type: (**A**) VK210a, without ANKKAEDA domain; (**B**) VK210 expressing ANKKAEA domain; (**C**) isolates VK247. Significant differences in haplotype frequency were observed among foci (χ^2^ = 29.0, *p* < 0.001). These differences remained significant among VK247 populations (χ^2^ = 37.9, *p* < 0.001).

An AMOVA test, performed using multilocus data from 159 *P. vivax* isolates, revealed that a greater variation occurred within malaria foci (73%) compared to between foci (27%) (*p* < 0.001). Haplotype frequencies were estimated per malaria focus, and categorized by their *pvcsp* type (Figure 5). VK210 parasites were further subdivided into those either expressing or lacking the ANKKAEDA domain at the carboxyl terminus (Figure 5A,B). Diverse pairings among *pvcsp* types, *pvs25* genotypes, and ribosomal variants were observed at different frequencies (Figure 5). *Pvcsp* VK247 was the most geographically widespread among the foci and exhibited all possible combinations with *pvs25* genotypes and ribosomal variants (Figure 5C).

Many haplotypes, specially the most frequent ones, were shared between two or more malaria foci, extending from northwestern to southern Mexico or vice versa. In the far northwestern (NWa) region, two highly frequent haplotypes were found, VK247_I-QT-rVar1 at 18.2% and VK247_I-QT-rVar2 at 63.6%, both present in Chihuahua and Sinaloa. They were also present in other foci, except in NWb and SMP, respectively. Haplotype VK247_I-QT-rV1 was at high frequency in OAX (42.1%), LR (38%), and SMP (15.4%). Meanwhile, VK247_I-QT-rV2 was also highly frequent in OAX (57.9%), NWb (18.9%), and LR (4.7%) (Figure 5). In the NWb focus, the haplotypes VK247_I-QT-rV2 (at 18.9%) and VK247_I-KT-rVar2 (at 48.6%) were detected in Durango, Nayarit, and Jalisco (NWb). The latter haplotype was also detected in LR at 2.4%. Another haplotype VK247_I-KT-rVar1 (24%), detected in NWb (Durango and Jalisco), was also detected in SMP at 30.8%. Of the two haplotypes detected in Oaxaca, one was shared with NWb and the other with SMP. The SMP and LR foci were the only ones that shared haplotype Vk210a-QI-rVar2, at 23.1% and 14.3%, respectively, and VK210b-QT-rVar1, at 15.3% and 2.3%, respectively. NWa, NWb, and LF foci had exclusive haplotypes as 210h-KT-rVar2 in Chihuahua, 247_I-QI-rVar2 in Durango and Nayarit, and VK247_II-QI-rVar2 in Nayarit. Several haplotypes in LR were exclusive (Figure 5). In LR, the VK247_II haplotype was found within three distinct multilocus haplotypes. In contrast, *P. vivax* from Nicaragua displayed high homogeneity, having only VK210a/e-QI-rVar2 haplotypes.

**Figure 6:**
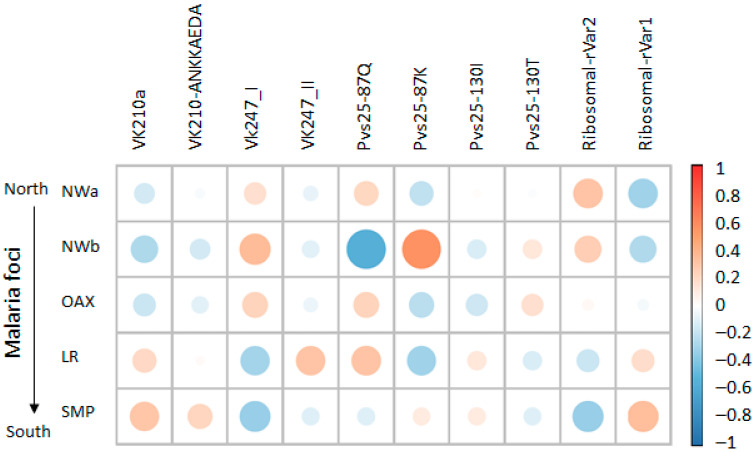
Heatmap of correlation patterns between genetic variants and *Plasmodium vivax* foci across Mexico. Coefficients range from −1 for strong negative correlation (shown in blue) to +1 for strong positive correlation (shown in orange), with 0 representing no association (white). Size of circles represents absolute value of correlation coefficient, with larger circles indicating stronger correlations (negative or positive), while smaller circles indicate weaker correlations closer to zero. Both displays include genetic variants and focus identifiers, revealing site-specific marker correlations and shared haplotype patterns among geographically proximal areas.

The Spearman correlation analysis revealed site-specific genetic variant associations and overlapping correlation patterns in geographically proximal foci (Figure 6). The SMP focus exhibited a positive association with both the VK210-ANKKAEDA and VK210a types. In contrast, the LR focus was primarily associated with VK210a. Malaria foci in the northwestern regions and Oaxaca were associated with VK247_I, whereas LR correlated with VK247_II. The strongest positive correlation was observed between LR and Pvs25–87K, followed by the VK247_I type with NWb, VK210a and ribosomal rVar1 with SMP, ribosomal rVar2 with NWa 1, and VK427_II and Pvs25 87Q with LR.

A linkage disequilibrium (LD) analysis was conducted by defining three *csp* types (VK210a, VK210-ANKKAEDA, and VK247), three Pvs25 haplotypes (QI, QT, KT), and two ribosomal variants (rVar2/rVar1) (Table 5). As anticipated [16], the southernmost Pacific population (SMP) exhibited the highest Association Index (*p* = 0.583). For the overall population, the I^A^_S_ value was low, and H0:VD = Ve was significant (*p* = 0.0004). Conversely, no evidence of LD was detected between *pvs25* genotypes and ribosomal variants within the nationwide VK247 population (*p* = 0.583). Similarly, LD between *pvs25* and the ribosomal variants was not evident when analyzing, independently, the VK210 and VK247 populations from LR (Table 5).

## 4. Discussion

In this study, *pvcsp*, *pvs25*, and the ribosomal polymorphism found in smear samples from various malaria foci (NWa, NWb, OAX, and LR) were consistent with previous findings in SMP [12,13,16,18]. All *pvcsp* types VK210a, VK210-ANKAEDA, and the conserved VK247_I; Pvs25 haplotypes QI, QT, and KT; and ribosomal variants were observed in SMP [13,16]. In SMP, a PCR-RFLP analysis of 379 samples showed that 48% were VK247_I, while 52% belonged to the VK210a group [12]. The VK247_I predominated in NWa/b and OAX foci and remain highly frequent in SMP and LR foci in Chiapas, and the presence of both VK210 types in NWa, SMP, and LR foci points to a history of substantial parasite circulation across Mexico before malaria transmission became focalized. In contrast, the lack of detection of VK247 and VK210-ANKKAEDA in Nicaragua might imply limited parasite exchange between Mexico and Central America beyond the immediate border areas.

VK210 parasites expressing the ANKKAEDA domain have been reported persistently in Middle East and Asia, for instance, in Vietnam [35] and Thailand [36], where its presence was attributed to parasite circulation across the Thai–Myanmar border. In Thailand, this polymorphic form was reported circulating in the 1990s [37]. In Myanmar, a country with high malaria transmission, *P. vivax* VK210 exhibited great diversity, as well as the presence of the ANKKAEDA domain and other diversified forms such as ANKKAENA and ANEAENA [38]. A recent resurgence of malaria cases in Pakistan revealed that all VK210 isolates were low-diverse and harbored the ANKKAEDA domain [39]. The authors attributed this to the high cross-border exchanges between Pakistan, Iran, and Afghanistan. In southern Iran, the presence of VK210-ANKKAEDA was reported decades earlier [40,41]. Similarly, VK247 parasites in Pakistan were identical to those reported earlier in Iran [40]. The *Pvcsp* VK247_1 found to be predominant in Mexico and across time in SMP [12] resembles a sequence from South America (isolated in 2008) and Iran [12,40]. This highlights the importance of molecular surveillance across time to understand genotype persistence and parasite evolution in regions with different eco-epidemiological conditions.

Pvs25 haplotype (QI, QT, and KT) frequencies varied among malaria foci in Mexico, likely due to specific ecological conditions, differences in vector species, and varying intensities of evolutionary forces. All Nicaraguan parasites had the Pvs25_QI haplotype, which matches strains from Central America and South America [13,29]. In South America, only the amino acid change Q87K has been reported across different regions (e.g., Brazil [42], Colombia, Peru [29], and Venezuela [43]), suggesting the presence of two genotypes: QI and KI. Beyond the Americas, the Q87K mutation has also been documented in Iran [44] and Mauritania [45]. In China, a different change (Q87L) was reported [46]. Pvs25 I130T polymorphism, which was found to be highly frequent in all malaria foci in Mexico, predominates in *P. vivax* populations outside Latin America, e.g., in China [46], Iran [44], Thailand [47], Myanmar [48], the Thai–Myanmar border [49], India [27,50], Bangladesh [51], and South Korea [52]. Additionally, two recent studies have indicated that the Pvs25 QT haplotype is highly frequent across Asia. A haplotype network analysis revealed that this haplotype might be more ancestral than the KT or QI haplotype, which likely diversified through distinct evolutionary routes from haplotype QT. Furthermore, any of these haplotypes might have given rise to the KI haplotype, most likely from QI in South America [47,48]. Using *pvs25* sequences, parasites from Central and South America clustered with those from Africa [53]. For *pvs25*, the highest nucleotide and haplotype diversity was detected in SMP, and the mismatch distribution SSD and Raggedness indexes suggest that SMP population has a complex demographic history.

In Mexico, the genetic characteristics *pvcsp* and *pvs25* suggest distinct introductions of *P. vivax* compared to those in Central or Southern America, with a limited circulation of some haplotypes across the continent. Parasite dispersal is influenced by compatibility and/or *P. vivax* adaptation to local vector species. Li et al. [54] found two ribosomal variants associated with vector infectivity. Parasites from Central America with the rVar2 variant produced high infections in *Ny. albimanus*, while parasites from other geographical sites expressing the rVar1 variant produced very low infection in this vector. The ribosomal rVar2 variant was at a higher frequency in northwestern foci, while rVar1 predominated in SMP and LR foci. In SMP, Pvs25 QI was found to be exclusively associated with ribosomal rVar2 and *pvcsp* VK210a, a haplotype found in the LR focus. Other haplotypes comprising any *pvcsp* type, Pvs25 QT/KT haplotypes, which combined exclusively with ribosomal rVar1, were highly infectious to *An. pseudopuntipennis* [16]. In SMP, these haplotypes were in linkage disequilibrium likely due to geographic and/or vector restriction.

Following the implementation of intensive control measures in the 1990s, malaria transmission became focalized [2]. The results from the AMOVA test, *F_ST_*, and Spearman correlation analysis using *pvs25* and/or multilocus analysis support the existence of a genetic structure among *P. vivax* populations in Mexico. The haplotype sharing among malaria foci and the *Nm* values using *pvs25* suggest that the genetic flow observed in Mexican *P. vivax* populations is a remnant of a past period, when malaria transmission was spread across the country. The persistence and evolution of parasites in each focus are likely due to their adaptation to local ecological conditions. This process may have led to the loss of haplotypes as the number of cases declined, a phenomenon previously documented in SMP [14,15]. The presence of several novel multilocus (CSP–Pvs25–ribosomal) haplotypes in malaria foci outside SMP was a notable finding. In northwestern (NWa and NWb) and OAX, *An. pseudopuntipennis* is the main malaria vector [24,55]. This species thrives in mountainous regions, with larvae commonly found along river margins amidst filamentous algae [24]. In these foci, the presence of several multilocus haplotypes suggests a high degree of adaptation of *An. pseudopuntipennis* to transmit a wide variety of *P. vivax* haplotypes (VK247/VK210a-QT-rVar2, VK247-QI/KT-rVar2, and VK210h-KT-rVar2), not reported in the SMP [16]. The multilocus LD encountered in *P. vivax* from Mexico suggests minimal recombination and a pattern of clonal transmission. However, the lack of linkage disequilibrium between the *pvs25* genotypes and the ribosomal variants within VK247 populations (nationwide, LR and NWb) suggests that free recombination happened in the past. These findings align with studies demonstrating that *An. pseudopunctipennis* from various regions in Mexico and Guatemala were highly susceptible to VK247 parasites from southern Mexico [26].

In the Lacandon region, both *An. pseudopunctipennis* and *Ny. albimanus* were reported as abundant [24]. Unlike in SMP, these vector species were found to be coexisting with other anopheline species and sharing breeding sites [24]. This region harbors numerous multilocus *P. vivax* haplotypes, and various were exclusive (e.g., VK247/VK210a-QI-rVar1, VK210b-QI-rVar2, VK210d-QT-rVar2). Conversely, low and non-significant I^A^_S_ values might suggest potential recombination events within the VK247 or VK210 population in LR and within the VK247 population in NWb. The possibility that these findings propose different vector specificities for the VK247 and VK210 populations warrants further investigation. The presence of VK210a-QI-rVar2 haplotypes in the LR and SMP foci and their predominance in Nicaragua (like Sal-I strain [56]) suggest that parasite exchange may be occurring between Mexico’s southern border and Central America.

In Mexico, the *Anopheles* genus includes 27 species, widely distributed across the country. LR has ecological conditions conducive to the year-round vector breeding of at least 11 species [24]. Of these, only three have been identified as primary malaria vectors: *An. pseudopunctipennis*, *Ny. albimanus*, and *Anopheles* (subgenus *Anopheles*) *vestitipennis* [57]. These species converge with other secondary vectors, including *Anopheles* (subgenus *Anopheles*) *punctimacula*, *Anopheles* (subgenus *Anopheles*) *apicimacula*, and *Anopheles* (subgenus *Nyssorhynchus*) *darlingi* [23,58]. In Brazil, mosquitoes *Ny. darlingi* transmits both VK210 [59,60] and VK247 parasites [61]. High parasite diversity is typically observed when numerous *Anopheles* species and high transmission intensity take place, such as in Brazil, Colombia, or Myanmar [23,62]. Vector control in the Lacandon region has been scarce or nonexistent due to a high level of insecurity, a lack of roads, and limited attention from the vector control program. Various indigenous ethnic groups have historically inhabited this geographic area. Between the 1970s and 1980s, the region experienced a significant migration of people from central and northern Mexico [63]. Additionally, people from Central American countries, primarily Guatemala, migrated to the area to flee violence, which created new conflicts with local residents and authorities over land ownership. Furthermore, the persistence of malaria in some regions in Mexico has been associated with several factors, including villages with poor infrastructure, reduced knowledge about malaria prevention, cultural factors, population movement, restricted access to primary health services, and environments with conflict [64,65,66].

This study represents the first genetic analysis of *P. vivax* from malaria foci outside SMP, demonstrating the feasibility of using smear samples for population-based studies. However, certain limitations must be considered, including potential bias from the non-uniform quality of the samples. Notwithstanding, all states contributed to the parasite diversity in NWa and NWb. In addition, several key findings were notable, e.g., the higher diversity in LR compared to other foci, the predominance of VK247 parasites in the northwestern foci and OAX, the high presence of VK210 parasites in LR, and the discovery of multiple novel multilocus haplotypes, among others.

*P. vivax* samples correspond to a period immediately following Mexico’s pre-elimination phase accreditation by the WHO in 2009 [3]. This parasite population might not entirely represent current genetic status of *P. vivax* in this country. However, when parasite transmission is mostly clonal, the highly frequent haplotypes would persist over time, as observed earlier in SMP [12,13,14,15]. These findings provide a baseline for parasite surveillance to evaluate persistent or emerging genotypes causing outbreaks in Mexico [2] or other regions in Latin America. In 2020, a total of 56 active microfoci were identified in 406 localities, as well as 71 residual foci in 320 localities [67]. This represents a powerful reminder that “malaria-free” status might not be permanent if receptivity and vulnerability to transmission remain. LR is currently the most challenging malaria focus [2], as it harbors the broadest reservoir of *P. vivax* haplotypes. This wide genetic diversity and high vector adaptation of *P. vivax* in different malaria foci might represent a significant challenge for malaria surveillance and elimination efforts.

## Figures and Tables

**Table 1:** Precedence and year of collection of smear blood samples randomly selected and diagnosed with *P. vivax* in Mexico.

State	Year of Collection		
	2010	2011	2012
Chihuahua	10	0	6
Sinaloa	20	20	19
Durango	5	4	36
Nayarit	5	16	23
Jalisco	19	0	6
Pochutla, Oaxaca	32	12	2
* Palenque and Ocosingo, Chiapas	32	20	48
Total smears	123	72	140

* Corresponds to the Lacandon region.

**Table 2:** *Pvcsp* genotype resolved by PCR-RFLP in parasites from different malaria foci in Mexico and from Nicaragua.

Malaria Foci:	Total (*n*)	Genotype by PCR-RFLP (*n*)									
		VK247			VK210						Mixed
		I	II	III	a	b	d	g	h	e	
NWa	28	26	-	-	1	-	-	-	1	-	-
NWb	64	62	-	1	-	-	-	-	-	-	VK210*a*/VK247_I
OAX	28	28	-	-	-	-	-	-	-	-	-
LR	63	28	6	-	20	5	2	1	-	-	VK210*a*/*b*
SMP **	39	18	-	-	15	6	-	-	-	-	-

*n*, number. Malaria foci: NWa (Chihuahua and Sinaloa); NWb (Nayarit, Jalisco, and Durango); OAX (Oaxaca); LR (Lacandon region). ** Results from previous study in SMP (southernmost Pacific in Chiapas) were included for comparison. Statistically significant difference was observed in VK210 and VK247 frequencies among malaria foci (χ^2^ = 35.8, *p* < 0.001).

**Table 3:** Parameters of diversity and neutrality tests in *P. vivax* among malaria foci in Mexico, using *pvs*25.

Malaria Foci	*n*	k	Hd ± SD	π ± SD	θ ± SD	Fu’s Fs	Tajima’s D	Mismatch SSD	Raggedness Index
NWa	23	0.504	0.467 ± 0.102	0.0021 ± 0.0005	0.0023 ± 0.002	−0.0717	−0.1312	0.014	0.1676
NWb	41	0.491	0.413 ± 0.081	0.0021 ± 0.0005	0.0020 ± 0.002	0.1917	0.0731	0.0008	0.1356
OAX	21	0	0	0	0	0	0	0	0
LR	44	0.433	0.414 ± 0.073	0.0018 ± 0.0004	0.0019 ± 0.001	0.0337	0.0889	0.0106	0.1779
SMP	39	0.837	0.667 ± 0.001	0.0035 ± 0.0003	0.002 ± 0.002	1.3887	1.4614	0.0175 *	0.1622 *
Overall	168	0.669	0.581 ± 0.026	0.0028 ± 0.0002	0.0015 ± 0.001	0.3085	1.2701	0.0086	0.1287

*n*, number of isolates; Hd, haplotype diversity; k, average number of nucleotide differences; *π*, nucleotide; and θ, genetic diversity. *Pvs25* is a fragment of 249 bp (nt 175–423). Two segregating sites and three haplotypes were detected in all populations, except in OAX, where only one haplotype was detected. * *p* < 0.05. Malaria foci NWa (Chihuahua and Sinaloa); NWb (Nayarit, Jalisco, and Durango); OAX (Oaxaca); LR (Lacandon region), and SMP (southernmost Pacific in Chiapas).

**Table 4:** *F_ST_* and *Nm* values of *P. vivax* between malaria foci in Mexico, using *pvs25*.

	NWa	NWb	OAX	LR	SMP
NWa		0.279	1.833	-	3.43
NWb	0.472 ***		0.151	0.227	1.019
OAX	0.120 *	0.623 ***		2.002	0.871
LR	0	0.524 ***	0.111 **		1.816
SMP	0.068	0.197 ***	0.223 ***	0.121 **	

*** *p* < 0.001; ** *p* < 0.01; * *p* < 0.05.

**Table 5:** Linkage disequilibrium analysis using loci: *pvcsp*, *pvs25*, and the ribosomal variants.

Data Set	*n*	Diversity Indexes		I^A^_S_	Var (VD)	(H0:VD = Ve)
		VD	Ve			*p* Value
Countrywide, three loci	159	0.8004	0.7315	0.047	0.002	0.0004
LR, three loci	43	1.0721	0.7451	0.219	0.0017	0.0001
SMP, three loci	39	1.1833	0.6865	0.362	0.001	0.0001
Countrywide, VK247 ^1^	120	0.496	0.4982	−0.002	-	0.583
NWb, VK247 ^1^	37	0.4215	0.4812	−0.062	0.0017	1.0
LR, VK247 ^1^	27	0.4317	0.4139	0.022	0.0034	0.135
Countrywide, VK210 ^1^	39	0.6506	0.4943	0.1594	0.0003	0.0004
LR, VK210 ^1^	16	0.4997	0.4889	0.0111	0.0012	0.566
SMP, VK210 ^1^	21	0.8937	0.4878	0.416	0.0011	0.0004

Three-marker haplotypes included the following: *pvcsp* types (VK210a, VK210-ANKKAEA, and VK247), *pvs25* (three polymorphic forms), and two ribosomal variants (rVar1 and rVar2). The index of Association (I^A^_S_). ^1^ The LD analysis between *pvs25* genotypes and ribosomal variants was estimated. *n*, number.

## Data Availability

Gene sequences were deposited in GenBank with accession numbers PX225108–PX225182 for pvcsp and PX236386–PX236514 for pvs25. All other relevant data are contained within the article or in the Supplementary Material.

## Supplementary Materials

The following supporting information can be downloaded at https://www.mdpi.com/article/10.3390/microorganisms13092221/s1, Figure S1: Concordance between *P. vivax* parasitemia, sample amount, and successful *pvcsp* gene amplification; Figure S2: PCR-RFLP of *pvcsp* gene from isolates in Mexico; Figure S3A: *Pvcsp* CRR polymorphism from malaria foci, Mexico; Figure S3B: *Pvcsp* nucleotide and amino acid carboxyl variable terminus in isolates from Mexico; Table S1: Percentage of samples with successful amplification of *pvcsp* gene, per geographical site and year, Mexico.

## Abbreviations

The following abbreviations were used in this manuscript:

*P. vivax*	*Plasmodium vivax*
CSP	Circumsporozoite protein
rRNA	Ribosomal RNA
PRM	Peptide repeat motif
CRR	Central repeat region
pvcsp	Circumsporozoite gene of P. vivax
RACCN	Autonomous Region of the North Caribbean Coast
PCR	Polymerase chain reaction
RFLP	Restriction fragment length polymorphism
AMOVA	Analysis of molecular variance
NWa	Far northwestern foci (Chihuahua, Sinaloa), Mexico
NWb	Northwestern foci (Nayarit, Durango, Jalisco), Mexico
OAX	Pochutla, Oaxaca, Mexico
SMP	Southernmost Pacific region in Chiapas, Mexico
LR	Lacandon region (Palenque, Ocosingo in Chiapas), Mexico
