# Supplementary material for: Population Dynamics of Plasmodium vivax in Mexico Determined by CSP, Pvs25, and SSU 18S rRNA S-Type Polymorphism Analyses
Source: Microorganisms. 2025 Sep 22;13(9):2221. doi: 10.3390/microorganisms13092221 (PMC12472771; doi:10.3390/microorganisms13092221)
Supplement: Supplementary file 1 [file microorganisms-13-02221-s001.zip › Table S1.pdf]

Table S1. Percentage of samples with successful amplification of the *pvcsp* gene, per geographical site and year, Mexico.

Malaria foci and sample origin	Year	*n*, total samples	n (%), samples with *pvcsp* amplification
NWa. Chihuahua-Sinaloa	2010	30	7 (25%)
	2011	20	7 (25%)
	2012	25	14 (50%)
	Sub total	75	28 (37%)
NWb. Nayarit-Jalisco-Durango	2010	29	10 (34%)
	2011	20	8 (40%)
	2012	65	46 (70%)
	Sub total	114	64 (56%)
OAX. Pochutla, Oaxaca	2010	32	19 (59%)
	2011	12	8 (66%)
	2012	2	1 (50%)
	Sub total	46	28 (60%)
LF. Palenque and Ocosingo, Chiapas.	2010	32	18 (56%)
	2011	20	12 (60%)
	2012	48	33 (68%)
	Sub total	100	63 (64%)
Total		335	183 (55%)

n, number.
