# Supplementary material for: Population Dynamics of Plasmodium vivax in Mexico Determined by CSP, Pvs25, and SSU 18S rRNA S-Type Polymorphism Analyses
Source: Microorganisms. 2025 Sep 22;13(9):2221. doi: 10.3390/microorganisms13092221 (PMC12472771; doi:10.3390/microorganisms13092221)
Supplement: Supplementary file 1 [file microorganisms-13-02221-s001.zip › Figure S1.pdf]

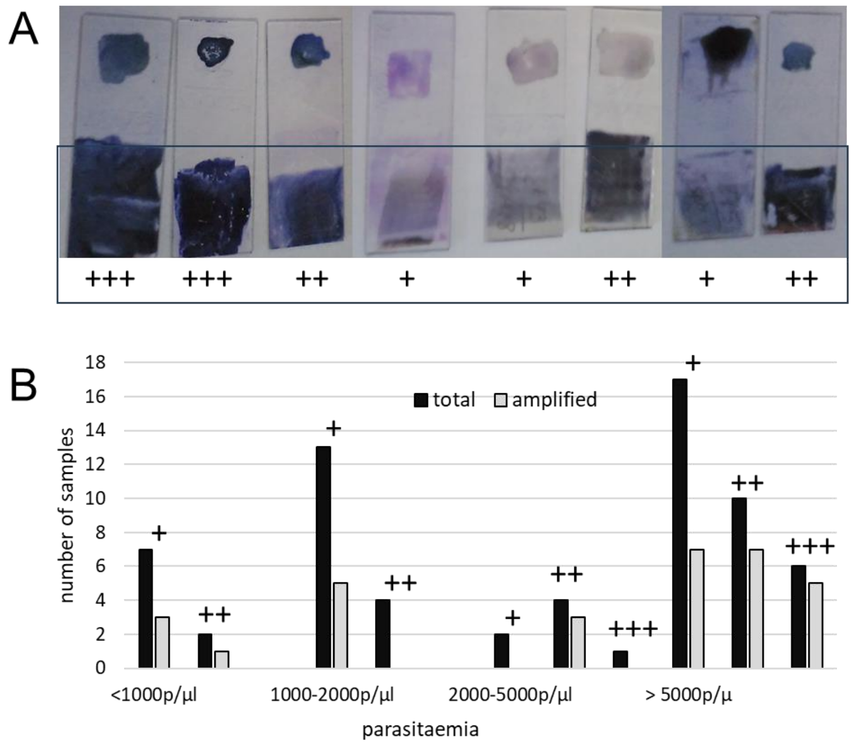

Figure S1. Concordance between *P. vivax* parasitemia, sample amount, and successful *pvcsp* gene amplification. Panel A shows *P. vivax*-infected samples derived from "thin" smears, along with their assigned visual scores: little (+), medium (++), and abundant (+++). This analysis included 66 samples originating from different malaria foci in Mexico. Notably, the thick smear portion was intentionally not scraped from these samples.

To assess the correlation between parasite densities or sample quantity and *pvcsp* gene amplification, a subset of 67 slides was randomly selected. For these slides, parasite counts were performed, and smear amount was visually estimated. Parasite density was quantified by observing 100 microscopy fields and recording both parasite and leukocyte numbers. Subsequently, 7,500 leukocytes per microliter was employed as a constant for calculating parasites per microliter.

In the analyzed smear samples, no significant correlation was observed between either parasitemia or smear quantity and *csp* gene amplification. The successful amplification varied considerably based on the state and year of sample collection. Notably, higher amplification success was achieved with samples from Oaxaca (60%) and Lacandon region of Chiapas (64%), while the Northwestern (MWa) focus exhibited the lowest rate (37%). This implies that the specific management, fixing, and preservation procedures implemented at each geographic site critically influenced DNA conservation.
