# Supplementary material for: Population Dynamics of Plasmodium vivax in Mexico Determined by CSP, Pvs25, and SSU 18S rRNA S-Type Polymorphism Analyses
Source: Microorganisms. 2025 Sep 22;13(9):2221. doi: 10.3390/microorganisms13092221 (PMC12472771; doi:10.3390/microorganisms13092221)
Supplement: Supplementary file 1 [file microorganisms-13-02221-s001.zip › Figure S2.pdf]

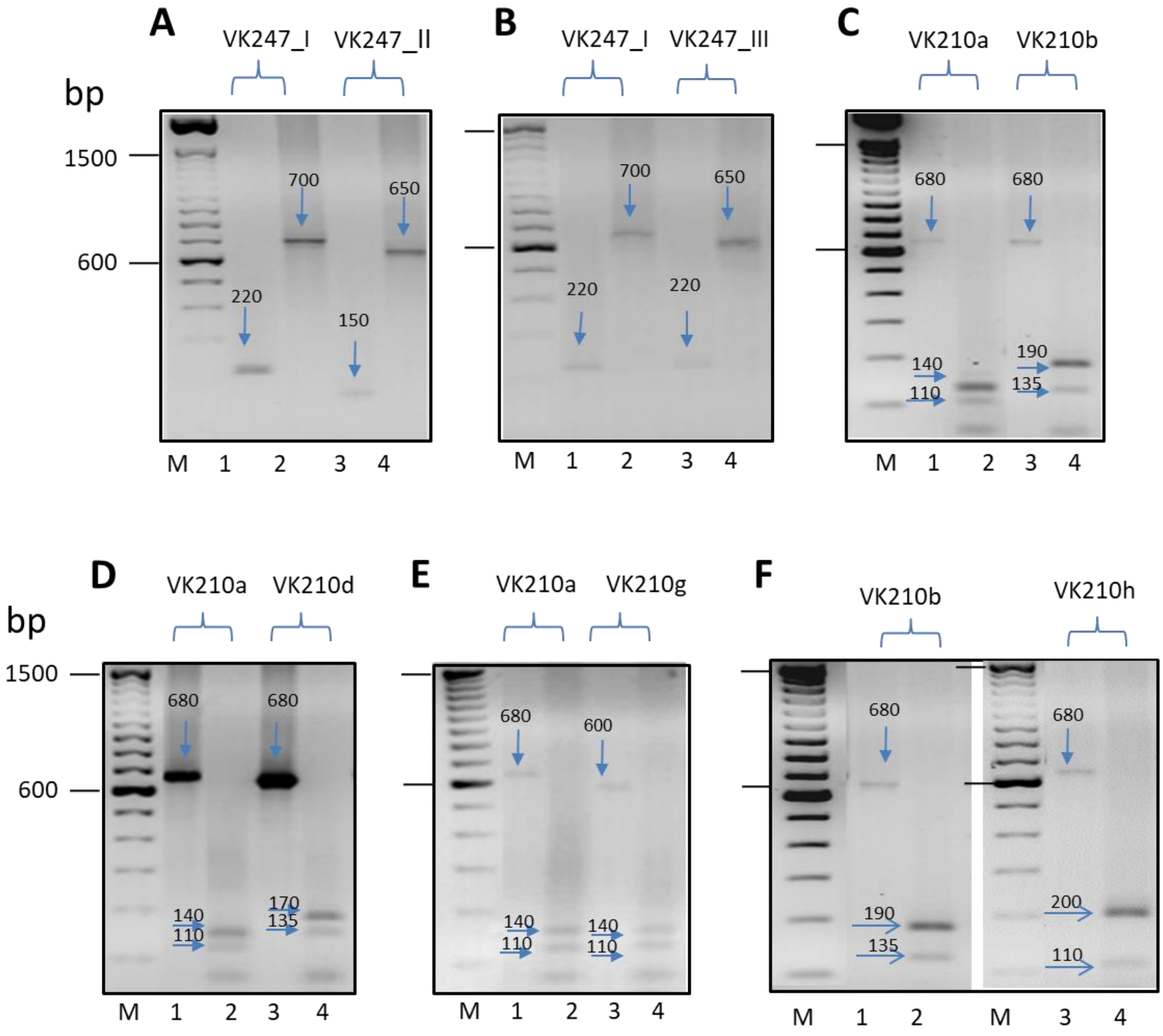

Figure S2. PCR-RFLP of *pvcsp* gene from isolates in Mexico. Panels A and B illustrate the comparison of VK247_II and VK247_III patterns *versus* VK247_I. Concurrently, Panels C-F present the patterns of VK210b, VK210d, VK210g, and VK210h compared to VK210a, respectively. bp, base pairs; M, molecular marker; lines 1 and 3 show digestion with BstOI and lines 2 and 4 with AluI. The numbers inside gels indicate the estimated bp for each band.
