# Supplementary material for: Population Dynamics of Plasmodium vivax in Mexico Determined by CSP, Pvs25, and SSU 18S rRNA S-Type Polymorphism Analyses
Source: Microorganisms. 2025 Sep 22;13(9):2221. doi: 10.3390/microorganisms13092221 (PMC12472771; doi:10.3390/microorganisms13092221)
Supplement: Supplementary file 1 [file microorganisms-13-02221-s001.zip › Figure S3A.pdf]

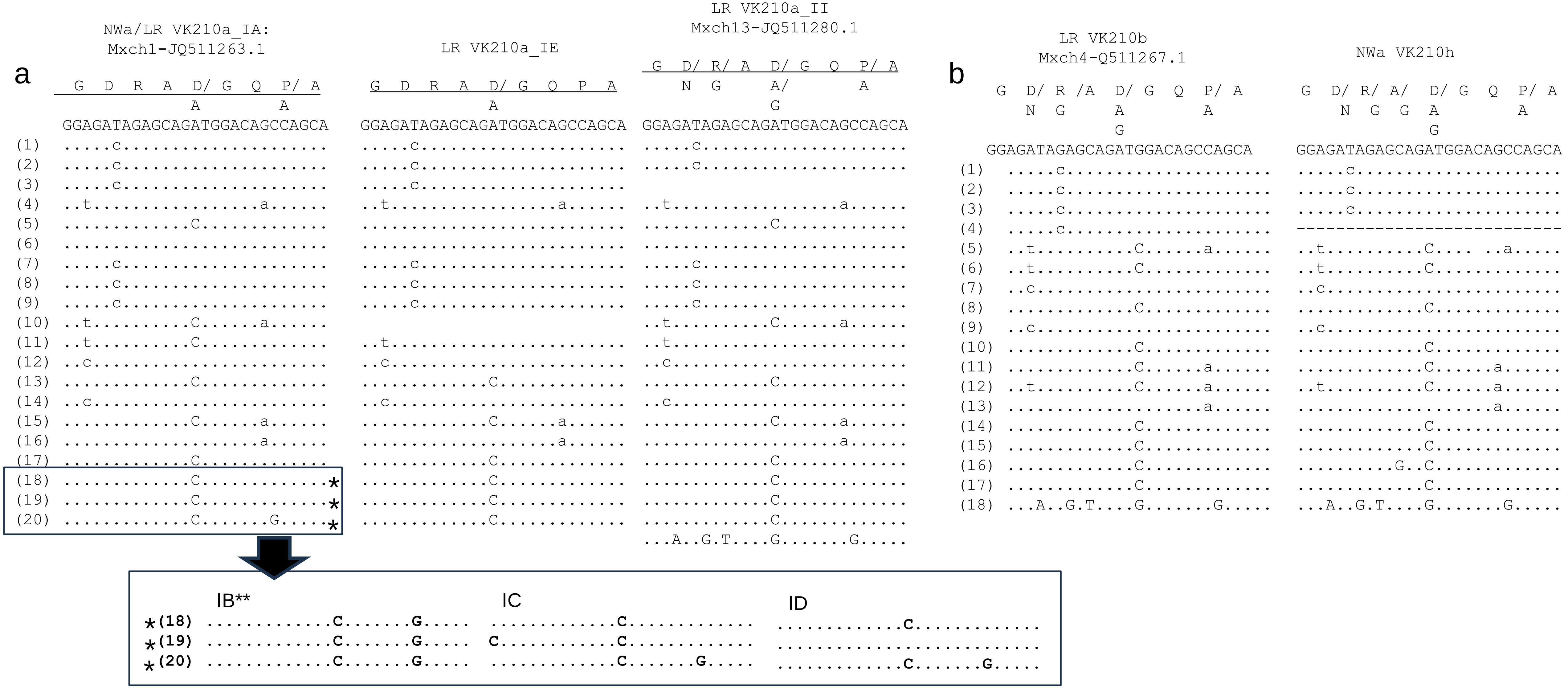

Figure S3A. *Pvcsp* CRR polymorphism from malaria foci, Mexico. a) The CRR of genotype VK210a_IA, other variants varied at last repeats: variants IB, IC and ID. VK210a_IE differed from variant IA (Mxch1) by a nonsynonymous mutations at one repeat and a deletion of one repeat unit. b) Comparison of CRR of genotype VK210b and variant VK210h, two mutations are shown. **this variant was similar to VK210a/e (JQ511285.1) from Nicaragua, except by the absence of one repeat unit in the middle of the CRR in IB variant. NWa, far Northwestern; LR, Lacandon region.
