# Supplementary material for: Population Dynamics of Plasmodium vivax in Mexico Determined by CSP, Pvs25, and SSU 18S rRNA S-Type Polymorphism Analyses
Source: Microorganisms. 2025 Sep 22;13(9):2221. doi: 10.3390/microorganisms13092221 (PMC12472771; doi:10.3390/microorganisms13092221)
Supplement: Supplementary file 1 [file microorganisms-13-02221-s001.zip › Figure S3B.pdf]

Amino acid:	G G N A	A N K K A E/G D A	G G N A	A N K K A E D A	G G N A	G G
Nucleotides:	ggaggaaacgca	gcaaacaagaaggcagaagacgca	ggaggaaacgca	gcaaacaagaaggcagaagacgca	ggaggaaacgca	Ggagga
VK210a (Sal-I)	************	************************	************	************************	....	......
Vk210b	........t..g	....	************	************************	3x	......
Vk210d	........t..g	....	************	************************	....	......
Vk210h	........t..g	....	........t..g	....	....	......
				G A G G Q A A G G N A A N K K A G D A		
				ggagcaggtggacaggcagcaggaggaaatgctgcaaacaaaaaggcaggagacgca		
Vk247_I, _III	........t..t	........a........G......	************	....		...C..
Vk247_II	........t..t	........a........G......	************	***		...C..

Figure S3B. *Pvcsp* nucleotide and amino acid carboxyl variable terminus in isolates from Mexico. Comparison of polymorphism between VK210h, VK210b and VK210d, and between VK247_I and _II. As reference VK210a (Sal-I, XM001614511.1), VK210b (Mxch4, JQ511267.1), VK210d (Mxch14, KF437876.1) and VK247_I (Mxch6, JQ511270.1) were used.
