# Supplementary figures and images for: Population Dynamics of Plasmodium vivax in Mexico Determined by CSP, Pvs25, and SSU 18S rRNA S-Type Polymorphism Analyses
Source: Microorganisms. 2025 Sep 22;13(9):2221. doi: 10.3390/microorganisms13092221 (PMC12472771; doi:10.3390/microorganisms13092221)

## Slide 1
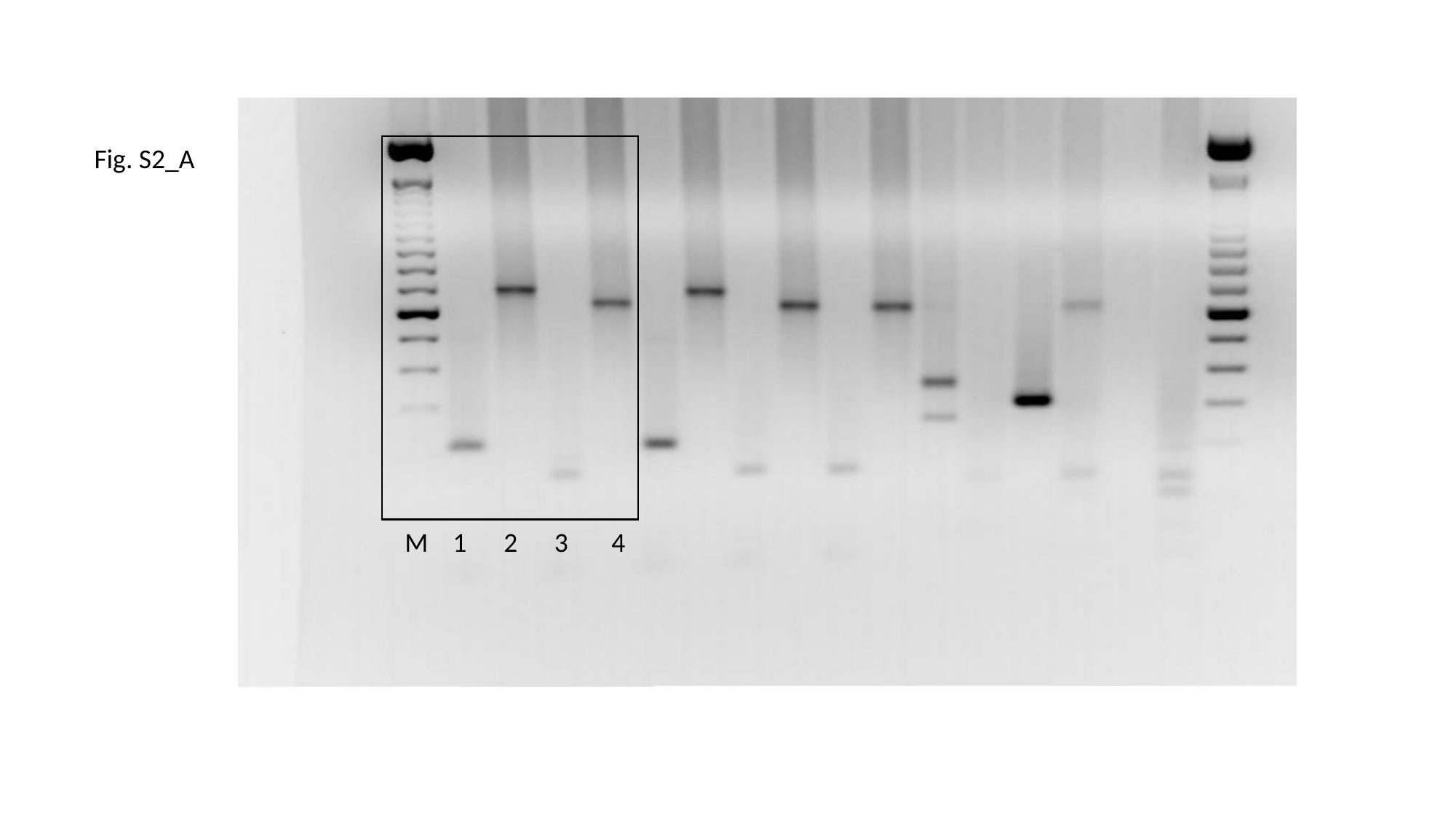

Fig. S2_A
M 1 2 3 4

## Slide 2
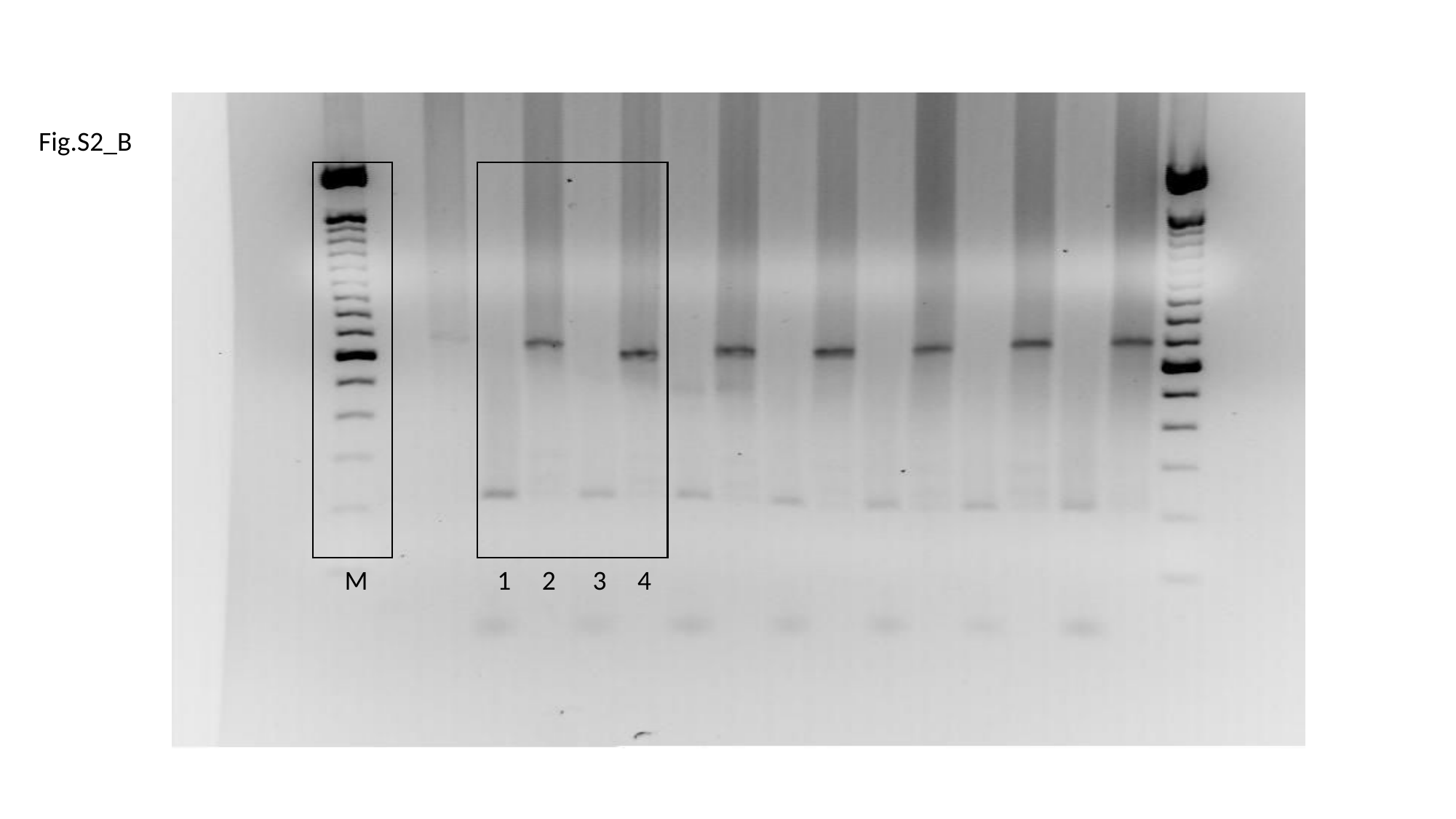

Fig.S2_B
M 1 2 3 4

## Slide 3
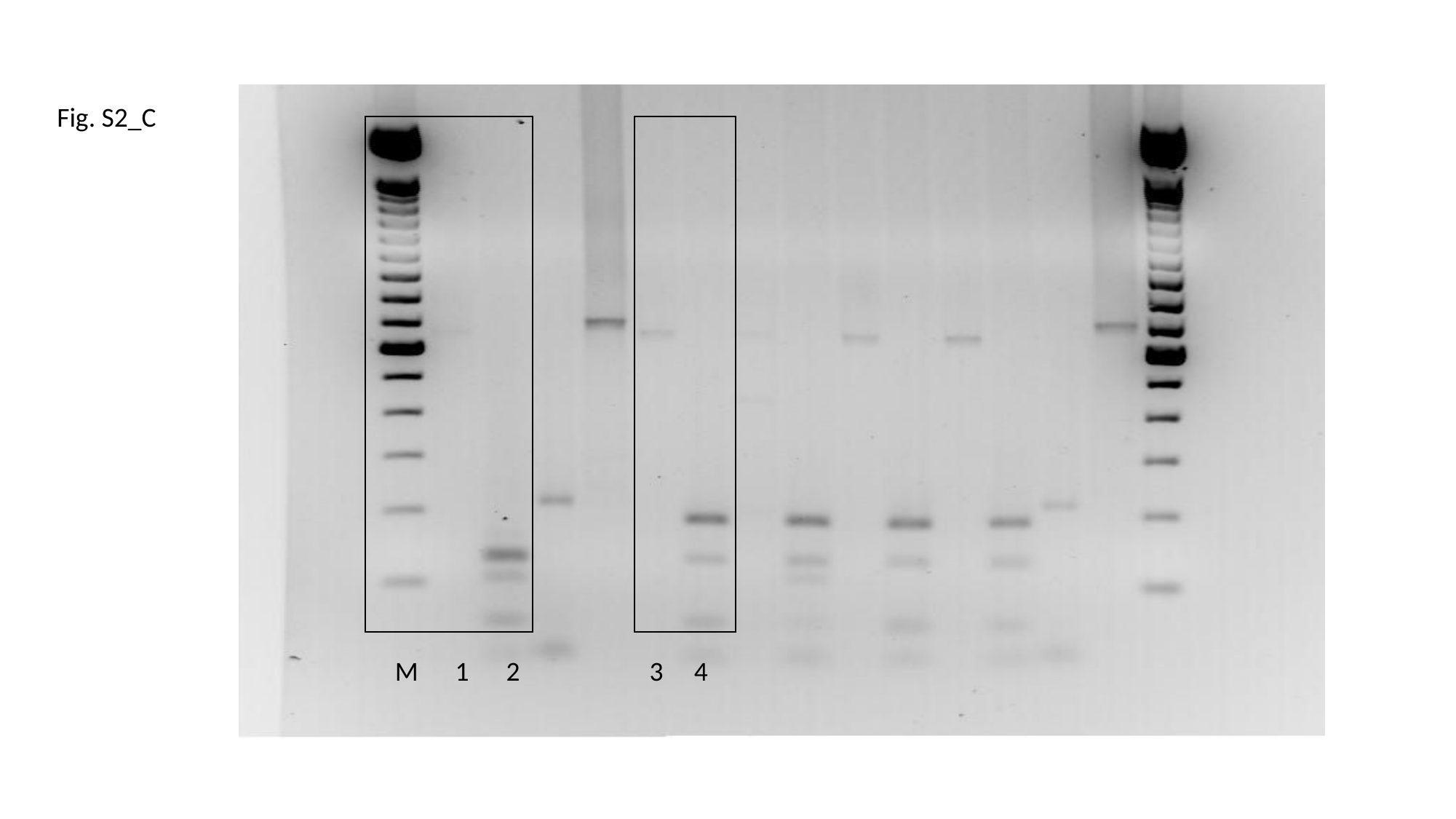

Fig. S2_C
M 1 2 3 4

## Slide 4
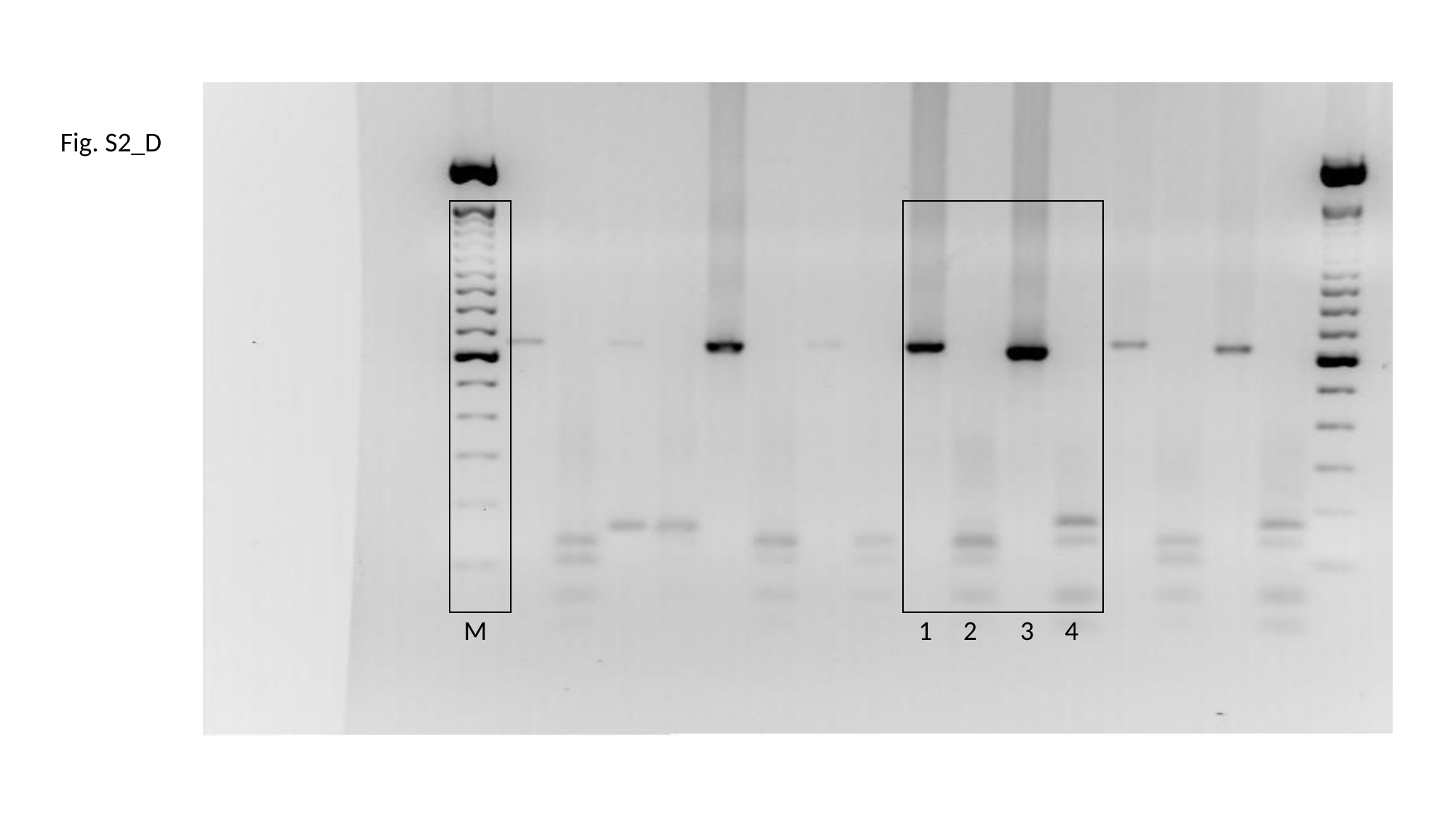

Fig. S2_D
M 1 2 3 4

## Slide 5
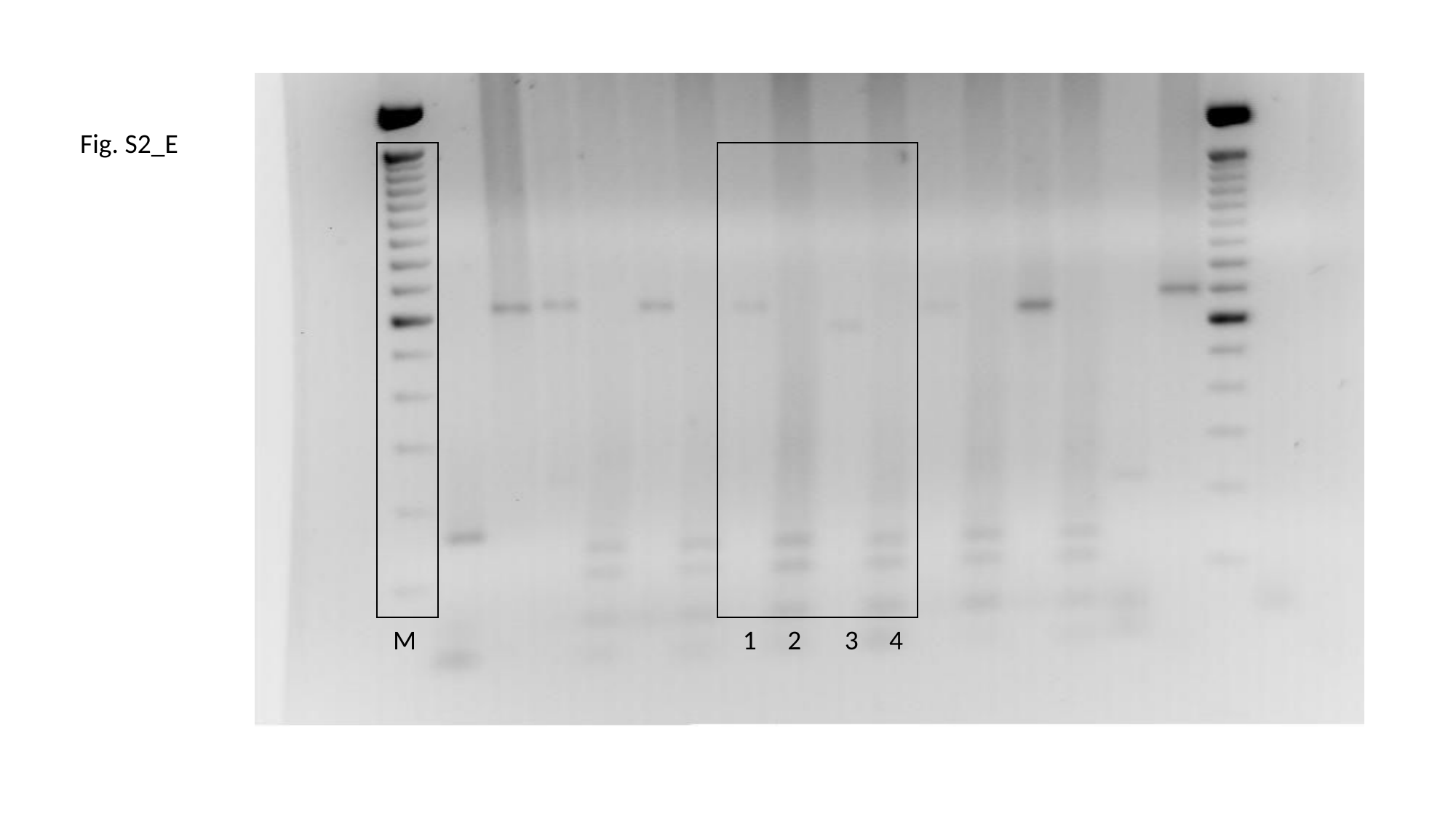

Fig. S2_E
M 1 2 3 4

## Slide 6
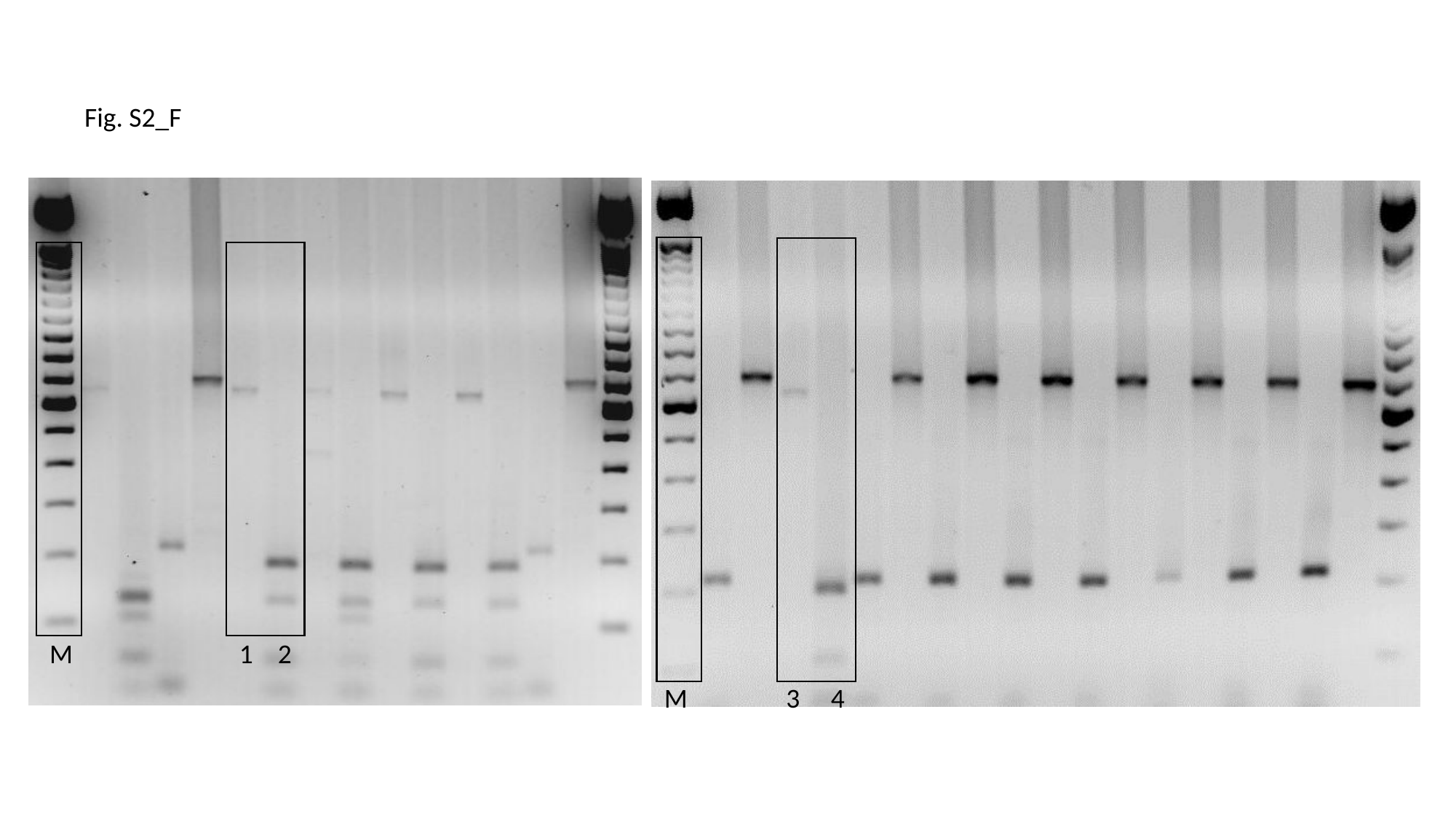

Fig. S2_F
M 1 2
M 3 4

Supplement: Supplementary file 1 [file microorganisms-13-02221-s001.zip › original gels of Figure S2 .pptx]
